# Brain ^18^F-FDG PET of SIV-infected macaques after treatment interruption or initiation

**DOI:** 10.1186/s12974-018-1244-z

**Published:** 2018-07-14

**Authors:** William Schreiber-Stainthorp, Sanhita Sinharay, Sharat Srinivasula, Swati Shah, Jing Wang, Lori Dodd, H. Clifford Lane, Michele Di Mascio, Dima A. Hammoud

**Affiliations:** 1 Center for Infectious Disease Imaging, Clinical Center, National Institutes of Health, Bethesda, MD USA; 2 Clinical Research Directorate/Clinical Monitoring Research Program, Leidos Biomedical Research, Inc., National Cancer Institute Campus at Frederick, Frederick, MD USA; 3 Biostatistics Research Branch, Division of Clinical Research, National Institute of Allergy and Infectious Diseases, National Institutes of Health, Rockville, MD USA; 4 Clinical and Molecular Retrovirology Section, Laboratory of Immunoregulation, National Institute of Allergy and Infectious Diseases, National Institutes of Health, Bethesda, MD USA; 5 Center for Infectious Diseases Imaging (CIDI), Clinical Center, National Institutes of Health (NIH), 10 Center Drive, Building 10, Room 1C-368, Bethesda, MD 20892 USA

**Keywords:** Antiretroviral therapy (ART), Fluorodeoxyglucose PET, SIV, Brain

## Abstract

**Background:**

Although rates of severe HIV-associated neurocognitive disorders have declined in the post-antiretroviral treatment (ART) era, subtle deficits persist, possibly exacerbated by treatment non-adherence. The actual effects of ART interruption/initiation on brain glucose metabolism as a reflection of viral replication and neuroinflammation remain unclear. Our study investigates how treatment initiation and interruption alter brain glucose metabolism in SIV-infected macaques, using ^18^F-FDG PET in correlation with plasma and CSF viral loads (VL) and cytokine levels.

**Methods:**

SIV-infected macaques (*n* = 7) underwent ART initiation only, ART interruption only, or both. Five uninfected animals served as controls. ^18^F-FDG PET imaging was performed at baseline and 1, 3, and 6 months after treatment modification. Mean and maximum standardized uptake values (SUV) for the whole-brain and subregions were calculated. Plasma and CSF VL and cytokine levels were measured. Paired *t* tests evaluated acute changes in whole-brain SUV from baseline to 1 month, while mixed-effect linear regression models evaluated changes over multiple timepoints and correlated SUV values with disease markers.

**Results:**

ART interruption was associated with increased SUVmean and SUVmax acutely, after 1 month (SUVmean 95% CI [0.044–0.786 g/ml], *p* = 0.037; SUVmax 95% CI [0.122–3.167 g/ml], *p* = 0.041). The correlation between SUV and time, however, was not significant when evaluated across all timepoints. Increased SUVmean and SUVmax correlated with decreased CD4+ and CD8+ T-cell counts and increased plasma VL. SUVmax was positively associated with increases in CSF VL, and there were borderline positive associations between SUVmax and IL-2, and between SUVmean and IL-15. The treatment initiation group showed no associations between imaging and disease biomarkers despite viral suppression, reduced cytokine levels, and increased CD4+ and CD8+ T-cell counts.

**Conclusions:**

ART interruption is associated with increased brain glucose metabolism within 1 month of treatment cessation, which, in concert with increased levels of pro-inflammatory cytokines in the CSF, may reflect neuroinflammation in the setting of viral rebound. Although we cannot assert neurologic damage in association with cerebral hypermetabolism, it is a concerning outcome of ART non-adherence. Treatment initiation, meanwhile, did not result in significant changes in brain metabolism. HIV-induced neuroinflammation may require a longer period to abate than our follow-up period allowed.

**Electronic supplementary material:**

The online version of this article (10.1186/s12974-018-1244-z) contains supplementary material, which is available to authorized users.

## Background

Since its introduction in the early 1990s, antiretroviral therapy (ART) has significantly decreased the mortality and morbidity and improved the quality of life for people living with HIV (PLWH) [1]. The effectiveness and availability of ART have improved over time: by 2015, 46% of the 36.7 million PLWH had access to ART [2, 3]. While ART is increasingly accessible, adherence to drug regimens depends on a number of factors and can be disrupted by phenomena including poverty, stigma, lack of social support, comorbid psychiatric disorders, and adverse drug effects including GI discomfort, lipodystrophy, renal dysfunction, and sensory neuropathy [4–10]. Due to these and other factors, ART adherence rates are estimated to be between 56 and 72%, with discipline declining over time, especially for populations with HIV-associated neurocognitive disorders (HAND) [7, 11–14].

Research into treatment nonadherence and interruption has documented various negative consequences, including rebounds in the cerebrospinal fluid (CSF) and plasma HIV viral load (VL), increased CSF neurofilament protein and neopterin, lymphocytic pleocytosis, greater likelihood of neurocognitive disorders, and elevated rates of disease and death when compared to continuous treatment [15–19]. Of note, despite the extensive evidence of benefit from ART, several studies have shown improvements in neuropsychological outcomes of PLWH after the interruption of ART treatment for periods as long as 96 weeks, adding nuance to our understanding of the effects of ART [16, 20, 21] and the balance between effects of HIV and side-effects of ART.

ART initiation generally suppresses HIV RNA in plasma and CSF, with the latter suppression being especially high for drugs with high central nervous system (CNS) penetration effectiveness (CPE) [22, 23]. High-CPE drugs, however, seem to have cognitive effects that are not fully understood. While some studies link them to improved neuropsychological outcomes, others have found a relationship between their use and an elevated risk of HIV dementia relative to drugs with low CPE [22–24]. Furthermore, while the current generation of ART has been effective in reducing rates of HIV dementia compared to the pre-ART era, milder forms of HAND persist [25]. Certain antiretrovirals (ARVs), such as efavirenz, have been specifically implicated as adversely affecting neurocognitive performance, while increased usage of others has been associated with a reduction in white matter volume when measured by magnetic resonance imaging (MRI) [26, 27]. The mechanisms by which ART may be causing toxicity are not fully understood, but various ARVs have been found to inhibit telomerase activity, induce oxidative stress, and interfere with mitochondrial enzymes [28–32].

In this study, we wanted to assess the longitudinal effects of ART initiation and interruption on brain glucose metabolism in a controlled setting. In doing so, we hoped to better understand how treatment interruption and resumption—whether medically sanctioned or non-prescribed—impact patients’ brains at the molecular level. Towards this goal, we longitudinally measured alterations in brain glucose metabolism, using positron emission tomography (PET) with ^18^F-Fluorodeoxyglucose (FDG), and correlated those with changes in CSF and plasma viral load, cytokines, and T lymphocyte counts as a result of treatment interruption and initiation. We anticipated that interrupting the treatment of simian immunodeficiency virus (SIV) infected monkeys would lead to increases in FDG uptake, CSF and plasma viral loads, and levels of inflammatory cytokines and to a decline in lymphocyte counts. We expected that the reverse would occur when treatment was initiated.

## Methods

### Animal welfare

All procedures were approved by the Animal Care and Use Committee of the National Institutes of Allergy and Infectious Diseases (NIAID), National Institute of Health.

### Animals, inoculation, and treatment

Twelve rhesus macaques (*Macaca mulatta*) were included in the study. Animals were either infected (*n* = 7, 5 females and 2 males, age = 6.44 ± 1.18 years, weight = 7.62 ± 2.13 kg) or healthy (*n* = 5, 3 males and 2 females, age = 7.63 ± 3.34 years, weight = 5.66 ± 0.77 kg). Animals that had been infected with either SIVmac251 (*n* = 4) or SIVE660 (*n* = 3) were separated into a treatment interruption or a treatment initiation group. The treatment initiation group (*n* = 5) had been infected for 467.6 ± 48.5 days without treatment. They were placed on an ART regimen consisting of daily subcutaneous injections of tenofivir (PMPA; 20 mg/kg) and emtricitabine (FTC; 30 mg/kg) with raltegravir (20 mg/kg) mixed with food twice per day and observed for 6 months afterwards with follow-up PET scans. The treatment interruption group consisted of three animals from the initiation cohort whose treatment was interrupted after an average of 255 ± 82 days of ART, and two animals who had treatment interruption after 570.5 ± 1.5 days of identical ART. The average duration of treatment prior to interruption for the all five animals in the interruption cohort was 381 ± 167 days. This group was also followed with PET imaging for 6 months after interruption (Additional file 1: Figure S1). The five uninfected animals were imaged once and served as controls.

### PET imaging

Baseline static FDG PET scans were completed before treatment modification (TM) with follow-up scans taking place from 1 to 6 months post-TM. Prior to each scan, animals were fasted for 12 h, then anesthetized with ketamine (10 mg/kg) and propofol (0.2 mg/kg), followed by 1.3–2% isoflurane. Animals were placed in a prone position in the scanner (GE Advance scanner or Siemens Biograph mCT PET/CT scanner). Each subject was injected with an intravenous bolus of FDG (5.06 ± 0.25 mCi; 187.03 ± 9.28 MBq), and after an approximately 60-min uptake period, a static scan was performed for 8 min through the brain. The respiration and heart rate of the subjects were monitored and stabilized by adjustments in anesthesia levels.

### Image analysis

Standardized uptake values (SUV) for the whole brain and 13 regions of interest (ROI) were measured using PMOD version 3.8 (PMOD Technologies Ltd., Zurich, Switzerland). PET emission scans were co-registered to a template MRI for which ROIs had been drawn in the caudate, putamen, pons, midbrain, prefrontal cortex, frontal cortex, anterior cingulate cortex, insula, temporal lobe, amygdala, hippocampus, thalamus, posterior cingulate cortex, and whole brain. Repeat analysis of whole brain uptake was performed using MIM for confirmation purposes (MIM, version 6.6; MIM Software Inc.).

### Viral load and cytokine concentrations

Viral load values were quantified in the plasma and CSF of each animal near the dates of the PET scans (mean time elapsed between scan and assay date = 8.2 ± 4.4 days). Cytokine levels in plasma and CSF were obtained using a MILLIPLEX _MAP_ Non-Human Primate Cytokine Magnetic Bead Panel (Millipore Sigma). Cytokines of particular interest included IL-2, IL-8, IL-10, IL-15, IL-1Ra, and MCP-1. All measurements were performed in duplicate and averaged across trials, except in the case of outlier values which were discarded.

### Statistical analysis

Paired *t* tests were performed for SUVmean and SUVmax between the baseline and 1 month post-TM timepoints. Mixed-effect linear regression models were fitted with SUVmax and SUVmean as outcomes and with time or biomarkers as the covariates. Random intercepts were assumed to account for within-subject correlations. These models were constructed separately for the interruption and initiation groups, in order to gauge whether associations between uptake and disease markers differed by type of treatment modification. Identical analyses were also performed with lumped data, by including animals from both treatment groups, to explore associations between brain metabolism and disease biomarkers irrespective of treatment status. Statistical significance was determined according to *p* values < 0.05. Direct adjustment for multiplicity (e.g., a Bonferroni adjustment) was not considered for a couple of reasons, including the limited power from small sample sizes typically used in non-human primate studies. Further, many of the hypothesis tests evaluated were on inter-related processes, making Bonferroni provide a conservative adjustment, particularly for an exploratory study.

## Results

### FDG uptake

#### SUVmean and SUVmax

##### Treatment interruption

**Fig. 1:**
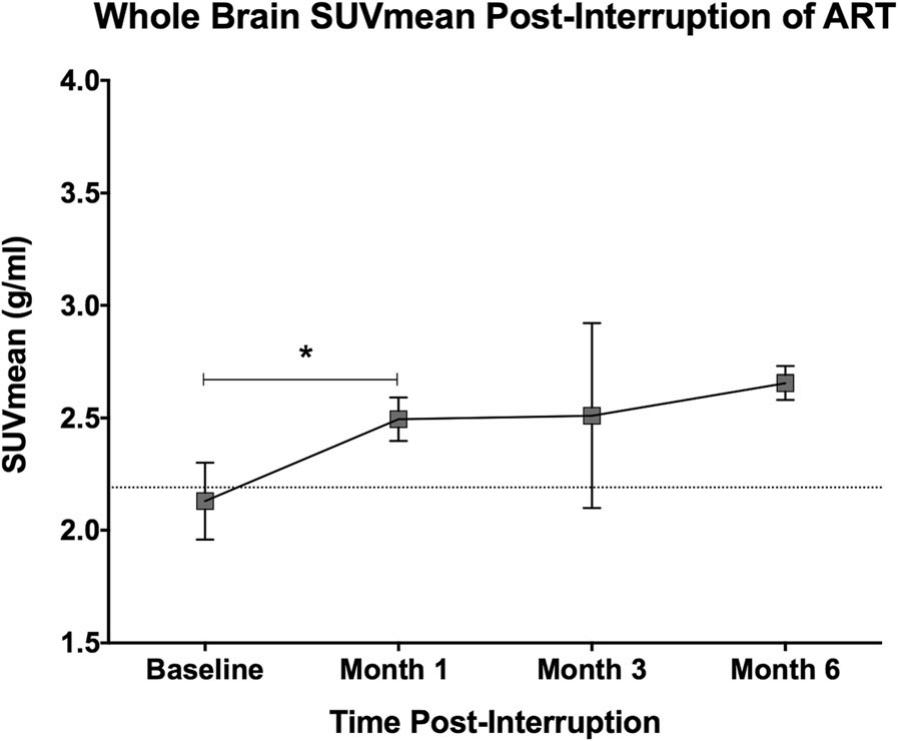
Average change in FDG SUVmean over time after the interruption of antiretroviral therapy. The dotted line represents the average SUVmean of uninfected healthy controls. Treatment interruption was associated with a significant increase in FDG SUVmean 1 month post-interruption (*p* = 0.037), but when all time points were considered in a mixed-effect linear regression model there was not a statistically significant effect of time (*p* = 0.236)

Average SUVmean and SUVmax values showed significant increases 1 month post-interruption (SUVmean: increase of 0.365 g/ml, or 17.1%, *p* = 0.037, *n* = 4; SUVmax: increase of 1.312 g/ml, or 23.9%, *p* = 0.041, *n* = 4; Fig. 1). At 3 and 6 months post-interruption, SUVmean and SUVmax values remained elevated over baseline. However, a mixed-effect linear regression model did not find a significant relationship between time and change in SUV values when all time points were considered.

##### Treatment initiation

**Fig. 2:**
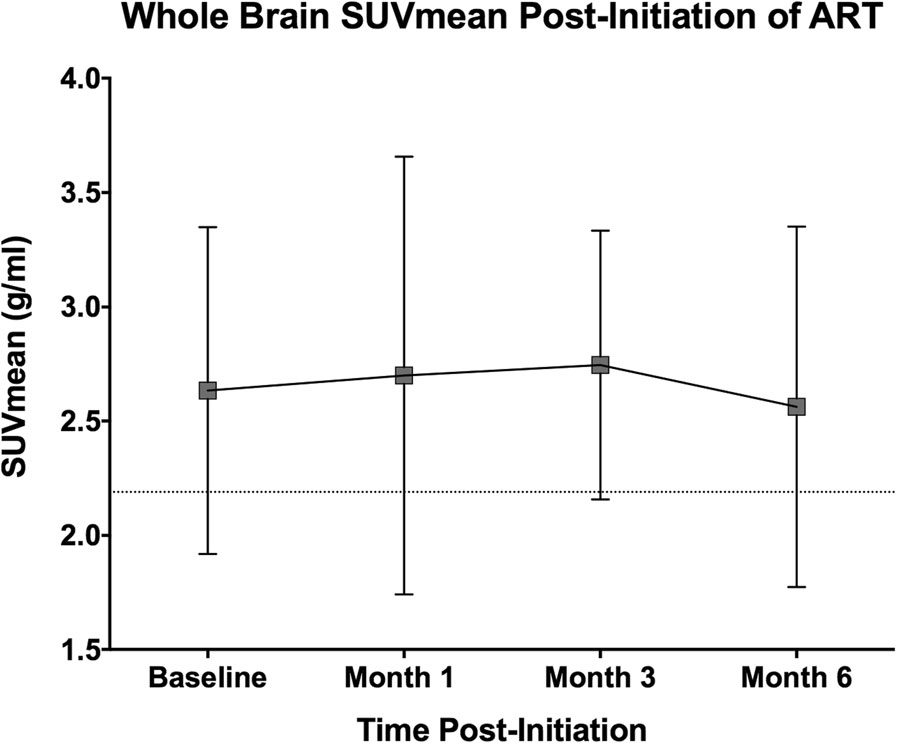
Average change in FDG SUVmean over time after the initiation of antiretroviral therapy. The dotted line represents the average SUVmean of uninfected healthy controls. At no timepoint did treatment initiation lead to statistically significant changes in uptake of FDG

Treatment initiation did not result in statistically significant changes in SUVmean or SUVmax at 1, 3, or 6 months post-TM (Fig. 2).

### Biomarkers of SIV replication/effects

#### Treatment interruption

Cessation of treatment was associated with an increase in CSF and plasma viral loads, reduced CD4+ and CD8+ T-cell counts, and reduced CD4/CD8 ratio (Table 1).

**Table 1:** Laboratory characteristics of animals before and after treatment modification

	Interruption				Initiation			
	Baseline	Month 1	Month 3	Month 6	Baseline	Month 1	Month 3	Month 6
CSF VL	0 (0)	272 (409)	16,741 (29,877)	327 (462)	6894 (10,533)	0 (0)	212 (473)	0 (0)
Log CSF VL	0 (0)	1.32 (1.54)	2.41 (2.29)	1.41 (1.99)	2.46 (2.56)	0 (0)	0.60 (1.35)	0 (0)
Plasma VL	3 (5)	79,070 (145,887)	258,608 (303,812)	919,845 (1,300,023)	362,834 (449,170)	41 (67)	20 (33)	8 (8)
Log plasma VL	0.27 (0.45)	4.11 (0.99)	4.10 (2.11)	4.52 (2.47)	4.89 (1.15)	1.15 (0.79)	0.79 (0.80)	0.65 (0.60)
CD4+ cells	783 (363)	576 (295)	450 (345)	526 (343)	235 (85)	487 (331)	444 (107)	648 (135)
Proliferating CD4+ cells	42 (22)	26 (7)	24 (16)	15 (13)	12 (5)	25 (9)	29 (16)	43 (17)
CD8+ cells	807 (424)	726 (385)	710 (427)	636 (350)	424 (256)	763 (483)	585 (415)	664 (258)
Proliferating CD8+ cells	29 (19)	36 (21)	61 (63)	38 (7)	24 (13)	35 (22)	28 (14)	40 (21)
CD4/CD8 ratio	0.98 (0.16)	0.83 (0.20)	0.67 (0.24)	0.80 (0.10)	0.64 (0.20)	0.70 (0.21)	0.93 (0.38)	1.08 (0.35)

Data are shown as mean values with standard deviations in parentheses

#### Treatment initiation

The introduction of treatment led to a decrease in CSF and plasma viral loads, increased CD4+ T-cell counts, and increased CD4/CD8 ratio (Table 1).

### Biomarkers of inflammation

#### Treatment interruption

Treatment interruption was associated with a general increase in CSF cytokines over the 6-month study period, but its impact on plasma cytokine levels was less consistent (Table 2).

**Table 2:** Plasma and CSF cytokines of the interruption and initiation cohorts over time

		Interruption				Initiation			
		Baseline	Month 1	Month 3	Month 6	Baseline	Month 1	Month 3	Month 6
Plasma	IL-2	5.51 (4.07)	4.25 (5.49)	5.75 (4.349)	2.62 (3.70)	9.10 (6.80)	5.63 (3.88)	5.82 (4.27)	5.85 (2.49)
	IL-10	2.98 (6.66)	2.98 (6.66)	2.98 (6.67)	3.56 (5.03)	8.07 (18.04)	0 (0)	1.22 (2.73)	0 (0)
	IL-15	5.14 (4.44)	4.67 (4.22)	5.55 (1.95)	3.98 (0.37)	7.14 (4.16)	3.94 (4.14)	4.98 (2.10)	4.83 (3.60)
	IL-8	25.59 (20.38)	26.65 (12.01)	77.25 (103.80)	22.61 (8.60)	144.60 (111.00)	18.70 (12.07)	38.39 (25.30)	20.10 (15.11)
	IL-1Ra	40.49 (58.85)	38.41 (53.41)	37.48 (42.52)	19.16 (9.24)	26.43 (14.19)	18.73 (9.73)	14.14 (8.96)	17.03 (13.65)
	MCP-1	240.3 (65.4)	217.9 (78.7)	204.4 (70.1)	245.5 (182.2)	349.5 (287.8)	266.2 (140.9)	254.1 (190.5)	270.4 (139.4)
CSF	IL-2	16.49 (10.89)	26.28 (23.52)	28.19 (6.96)	32.87 (12.38)	21.25 (14.25)	19.49 (6.29)	23.49 (6.20)	22.81 (8.09)
	IL-10	20.89 (13.37)	40.67 (31.34)	35.61 (8.29)	43.68 (18.27)	29.83 (17.83)	33.63 (13.12)	34.61 (6.65)	28.72 (15.97)
	IL-15	14.65 (2.93)	16.14 (3.64)	16.54 (5.01)	21.37 (1.65)	19.58 (3.01)	16.83 (2.90)	14.60 (1.53)	18.24 (4.49)
	IL-8	10.50 (3.71)	21.23 (21.2)	14.84 (3.40)	25.1 (0.17)	13.61 (4.30)	8.80 (6.58)	10.43 (4.13)	7.90 (2.22)
	IL-1Ra	0.92 (1.85)	5.39 (6.30)	7.13 (4.55)	8.97 (2.73)	8.27 (1.65)	2.20 (4.39)	3.19 (5.53)	1.76 (3.05)
	MCP-1	446.7 (76.0)	947.2 (1070.0)	617.1 (153.7)	702.8 (303.5)	641.1 (141.4)	422.2 (121.1)	526.6 (137.6)	481.0 (127.6)

Data are shown as mean values with standard deviations in parentheses

#### Treatment initiation

CSF levels of IL-8, IL-1Ra, and MCP-1 decreased in the wake of treatment initiation. Plasma cytokine levels decreased to an even greater degree, particularly IL-8 and MCP-1 (Table 2).

### Correlations between laboratory parameters and FDG uptake

#### Lumped data

When longitudinal data were combined, irrespective of treatment status, there was a statistically significant negative correlation between SUVmean and CD4+ T-cell counts (parameter estimate = − 0.001, *p* = 0.024) and suggestions of negative correlation between SUVmax and CD4+ T-cell count (paramater estimate = − 0.002, *p* = 0.053) and between SUVmean and CD8+ T-cell count (parameter estimate = − 0.001, *p* = 0.06).

#### Treatment interruption

##### SUVmax

For the treatment interruption cohort, there were significant positive correlations between maximum FDG uptake and log10 CSF viral load (parameter estimate = 0.411, *p* = 0.023) as well as log10 plasma viral load (parameter estimate = 0.272, *p* = 0.038), and significant negative correlations between maximum FDG uptake and CD4+ T-cell counts (parameter estimate = − 0.002, *p* = 0.028) as well as CD8+ T-cell counts (parameter estimate = − 0.002, *p* = 0.022). There was a suggestion of a positive correlation between SUVmax and CSF IL-2 (parameter estimate = 0.045, *p* = 0.052). Correlations are summarized in Table 3.

**Table 3:** Correlations between FDG uptake and markers of disease post-treatment interruption

	SUVmax	SUVmean
Length of interruption	NS	NS
Log10 CSF viral load	0.411; *p* = 0.023	NS
Log10 plasma viral load	0.272; *p* = 0.038	0.068; *p* = 0.052
CD4+ cell count	− 0.002; *p* = 0.028	− 0.001; *p* = 0.01
Proliferating CD4+ cell count	NS	− 0.010; *p* = 0.033
CD8+ cell count	− 0.002; *p* = 0.022	− 0.001; *p* = 0.022
Proliferating CD8+ cell count	NS	NS
CSF IL-2	0.045; *p* = 0.052	NS
CSF IL-15	NS	0.043; p = 0.048

Data are reported as slope coefficients and *p* values for each regression analysis

##### SUVmean

There was a trend towards a positive correlation between mean FDG uptake and CSF IL-15 (parameter estimate = 0.043, *p* = 0.048), and between mean FDG uptake and log10 plasma viral load (parameter estimate = 0.068, *p* = 0.052). There were significant negative correlations between mean FDG uptake and CD4+ T-cell counts (parameter estimate = − 0.001, *p* = 0.01), proliferating CD4+ T-cell counts (parameter estimate = − 0.01, *p* = 0.033), and CD8+ T-cell counts (parameter estimate = − 0.001, *p* = 0.022). Correlations are summarized in Table 3.

#### Treatment initiation condition

After treatment initiation, there were no significant correlations between maximum or mean FDG uptake and individual biomarkers.

### Subcompartmental data

**Fig. 3:**
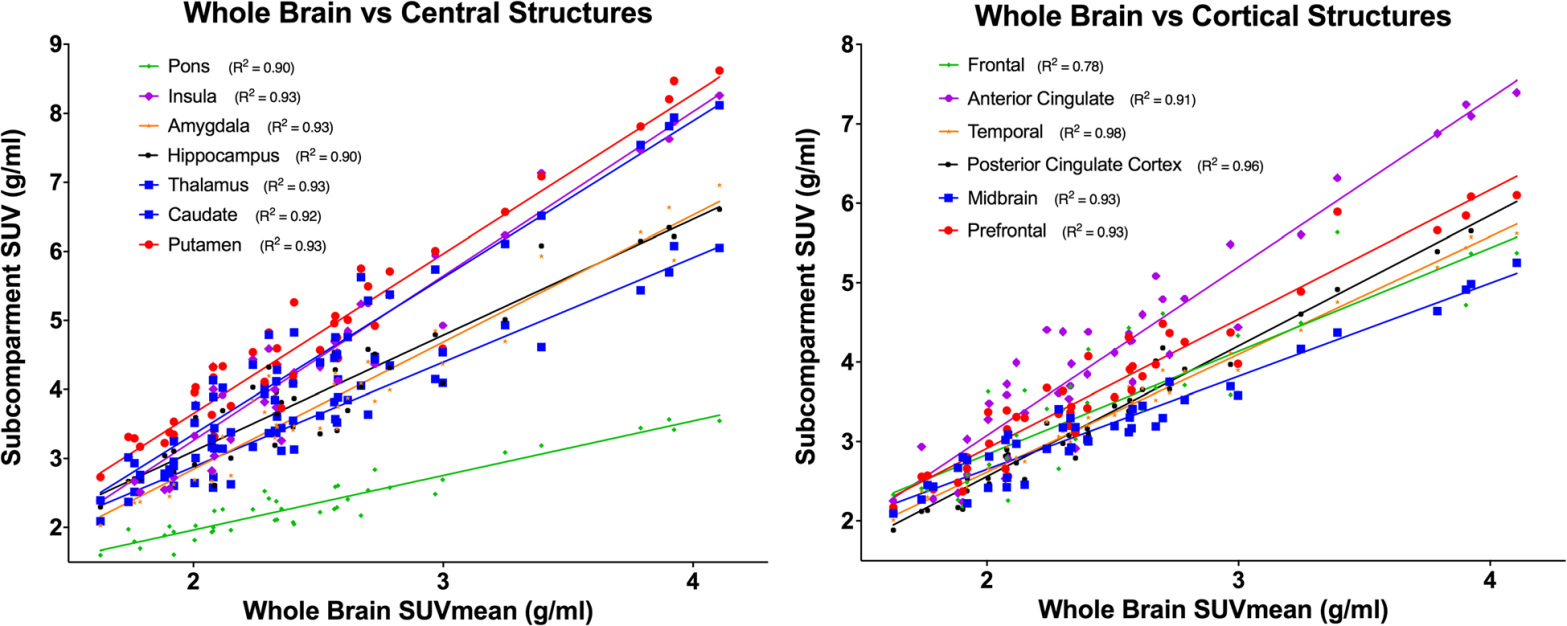
Correlations between whole brain SUVmean and subcompartmental SUVmean. Both central (left) and cortical (right) structures were compared to the whole brain. Correlation *R*^2^ values are reported for each subcompartment. We did not identify regional predilections for FDG changes

There were strong correlations in mean FDG uptake for the whole brain and all subcompartments. The lowest correlation was between the orbital prefrontal (frontal pole) region and the whole brain, with an *R*^2^ value of 0.78 (Fig. 3).

## Discussion

The current study has clearly demonstrated that discontinuation of ART is associated with increased metabolic activity in the brain as measured by FDG uptake, as early as 1 month after interruption. This is likely due to neuroinflammation secondary to SIV replication and is extremely relevant to patients taking “drug holidays” and to investigational treatment interruption studies. The interruption-induced increases in brain metabolism observed in our experiment were significantly correlated with increased CSF and plasma VL and cytokines and decreased CD4+ and CD8+ T-cell counts.

The concept that glucose metabolism reflects disease activity in HIV/SIV is not new. Although not necessarily concentrating on the brain, previous imaging studies have correlated HIV laboratory and clinical biomarkers to FDG PET data in the periphery: FDG uptake in the lymph nodes, for example, has been correlated with viremia in both treated and untreated patients, and inversely correlated with CD4+ T-cell counts [33–35]. Another study performed with SIV-infected monkeys found that tissues with greater FDG uptake, such as the ileocecal lymph nodes, exhibited more productive SIV infection as measured by SIV RNA than did other lymph nodes with lower levels of FDG uptake [36]. Lymph node FDG uptake has also been found to correlate with immunological variables, including percentage of CD8+, CD38+, and RO+ T cells in untreated patients [37]. In the brain, in the pre-ART era, basal ganglia hypermetabolism was described as occurring in the early stages of infection, with eventual transition to cortical and subcortical hypometabolism in more chronic stages of the disease [38–40]. This early-stage hypermetabolism was linked to worsened neuropsychological performance and was found in groups of patients with low CD4+ T-cell counts [41, 42]. Our data resemble those findings in that we show hypermetabolism in the setting of ART interruption and viral rebound, though the regional variations described above were not seen in our study, as evidenced by the strong agreement between whole brain and subcompartment uptake of FDG in this study (Fig. 3). This could be due to the short duration of follow-up we had (6 months). Our data, nonetheless, extend previous findings by correlating hypermetabolism in the brain with markers of disease course, most notably drops in CD4+ and CD8+ T-cell counts and increases in plasma and CSF viral loads.

Although we cannot assert neurologic damage in association with increased cerebral metabolism post-interruption, the phenomenon raises concerns about the potential effects of even brief periods of non-adherence to ART. FDG, while not a specific marker of inflammation, often correlates with the neuroinflammatory burden [43]. The hypermetabolism observed in this study, therefore, is likely the result of neuroinflammation in the setting of viral rebound. This possibility is supported by the positive correlations between post-interruption FDG uptake and levels of the CSF cytokines IL-2 and IL-15. Both IL-2 and IL-15 are pro-inflammatory cytokines which share two signaling subunits while having distinct functionality: IL-2 induces T cell, B cell, and NK cell proliferation and cytokine release while IL-15 causes increased activation of CD8+ T-cells [44–49]. Levels of IL-2 have been found to be higher in patients with higher viral loads and those with higher CD4+ T-cell counts, while levels of IL-15 have been correlated with higher viral loads and lower CD4+ T-cell counts [50, 51]. The increased levels of these cytokines post-interruption therefore suggest an inflammatory response that is occurring in parallel to increased FDG uptake. The correlation of FDG uptake with CSF, but not plasma, concentrations of IL-2 and IL-15 further suggests that the CNS response may be somewhat disconnected from the peripheral response, and that peripheral measurements may not adequately represent cerebral disease course. Interestingly, there was no further correlation between time post-treatment interruption and change in SUV values beyond the first month of treatment cessation. These data indicate that the effects of interruption on glucose metabolism specifically might be most intense in the acute phase, and stabilize afterward.

The results observed in the treatment initiation group were more heterogeneous than those from interruption. Post-initiation, some animals showed decreases in brain metabolism at months one and three, and there was an average decrease in brain metabolism at month six, but these changes were not consistent or strong enough to reach statistical significance. This was despite a marked reduction, within 1 month, of plasma and CSF viral loads, an increase in CD4+ and CD8+ T-cell counts, and lowered levels of CSF cytokines including IL-1Ra and MCP-1, similar to what has been described previously in association with ART initiation [52–56]. The lack of consistent changes in brain metabolism in the wake of treatment initiation and improved measures of disease course may suggest that the abatement or decrease of HIV/SIV-related neuroinflammation requires a longer period than our study spanned. Changes in brain chemistry and function are likely to lag behind changes of conventional disease measures like CD4+ T-cell counts and viral load. This pattern has been shown in other imaging modalities: a magnetic resonance spectroscopy study of ART initiation found that while 3 months of treatment improved the clinical biomarkers of HIV+ patients, levels of brain metabolites that measure brain injury remained abnormal [57]. Other PET studies have more directly monitored cerebral inflammation post-treatment: two groups assessed cerebral levels of the translocator protein (TSPO), a marker of microglial inflammation, finding elevations in TSPO binding for HIV+ individuals even when the subjects were optimally treated and neurocognitively asymptomatic [58–60]. Interestingly, one of those studies failed to find an association between TSPO binding and levels of CSF cytokines including eotaxin, MIP-1β, IL-8, and IL-10 [61–64]. This finding is notable, as our own experiment failed to find a correlation between FDG uptake and cytokine levels post-initiation, and suggests that treatment with ART may result in an unlinking of these variables under certain circumstances.

Our data should be interpreted with some qualifications. Our sample size is relatively small. The subjects, even within a single cohort, were relatively heterogeneous in terms of baseline cytokine levels which could have played a role in their subsequent reaction to treatment and/or treatment removal. While treatment regimens were carefully considered, one antiretroviral, Raltegravir, was mixed in with food and its intake could be affected by the animal’s eating habits, especially during sickness. Future experiments might address some of these issues, as well as incorporate longer follow-up periods and more frequent sampling, especially within the first month of treatment modification when the most dramatic changes tend to occur. Protocols might also seek to incorporate interruption and initiation guidelines which more closely resemble those used clinically, such as CD4+ T-cell count thresholds or viral load limits that are tied to treatment resumption. For a more direct assessment of inflammation, the usage of radioligands that bind directly to inflammatory markers like the translocator protein (TSPO), a mitochondrial membrane receptor known to be upregulated in activated microglia, would offer an additional perspective on inflammatory changes post-interruption and initiation of ART.

## Conclusions

Our study showed that treatment interruption was associated with rapid increases in brain metabolism along with disease intensification, with correlations between imaging and laboratory data. We also showed that treatment initiation, while improving biomarkers of disease, does not moderate pre-treatment brain metabolism within the six-month time period studied. Based on our findings, we believe that even brief gaps in treatment can potentially lead to negative outcomes, both molecularly and functionally. As a whole, our data support modern treatment guidelines, which advocate treatment as early as possible and maximal adherence to ART regimens [65].

## Additional file

Additional file 1: **Figure S1.** Experimental design diagram for the initiation and interruption cohorts. (TIFF 9496 kb)

## Abbreviations

ART: Antiretroviral therapy
ARV: Antiretroviral
CNS: Central nervous system
CPE: CNS penetration effectiveness
CSF: Cerebrospinal fluid
FDG: Fluorodeoxyglucose
HAND: HIV-associated neurocognitive disorders
HIV: Human immunodeficiency virus
MRI: Magnetic resonance imaging
PET: Positron emission tomography
PLWH: People living with HIV
SIV: Simian immunodeficiency virus
SUV: Standardized uptake value
TM: Treatment modification
TSPO: Translocator protein
VL: Viral load
