# Supplementary figures and images for: Brain 18F-FDG PET of SIV-infected macaques after treatment interruption or initiation
Source: J Neuroinflammation. 2018 Jul 14;15:207. doi: 10.1186/s12974-018-1244-z (PMC6046092; doi:10.1186/s12974-018-1244-z)

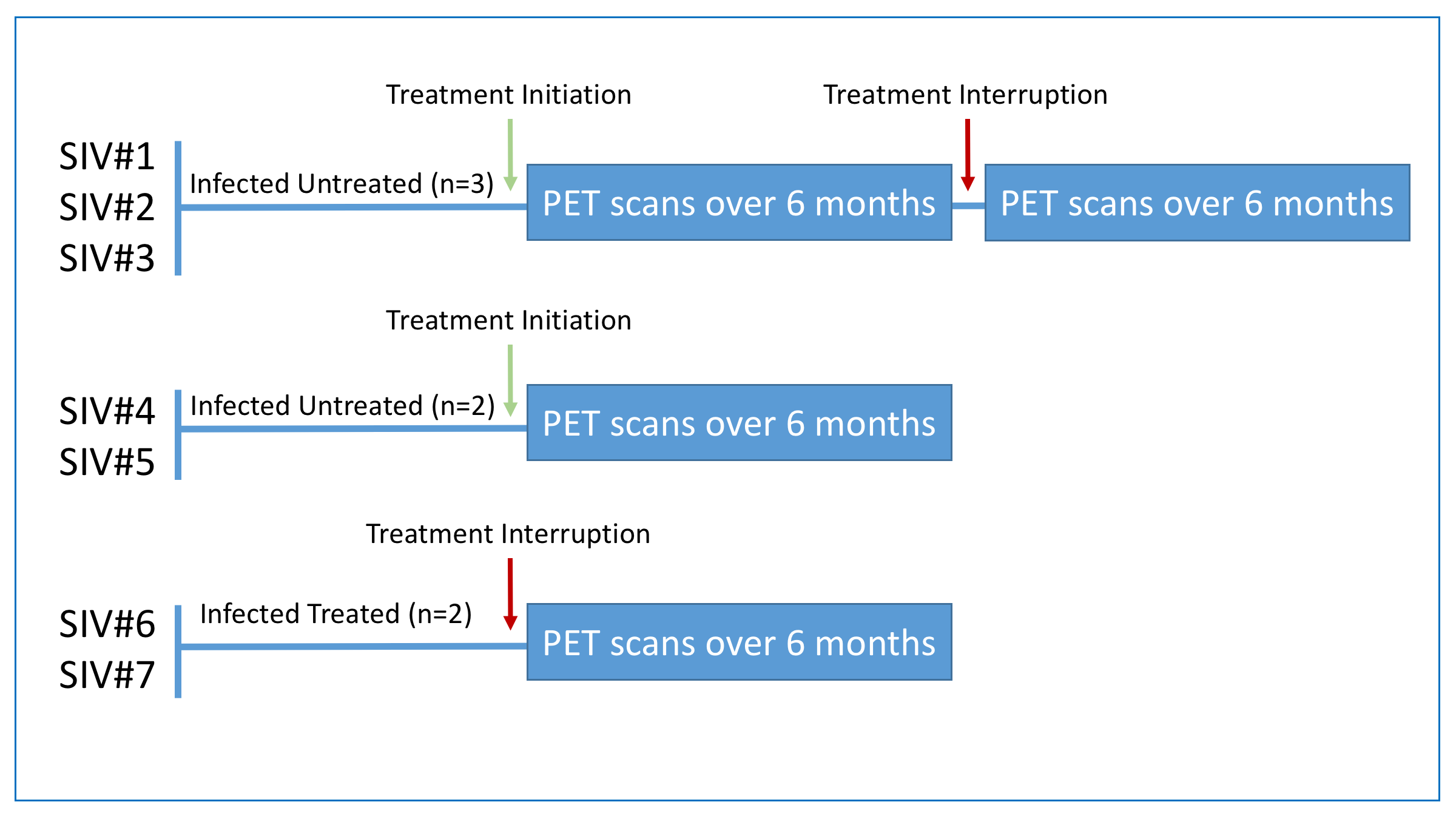

Supplement: Supplementary file 1 — Figure S1. Experimental design diagram for the initiation and interruption cohorts. (TIFF 9496 kb) [file 12974_2018_1244_MOESM1_ESM.tiff]
